# Multidimensional Geriatric Assessment with MAGIC Questionnaire and Quality of Life in Elderly Primary Care Patients

**DOI:** 10.3390/ijerph17197089

**Published:** 2020-09-28

**Authors:** Fátima Dios-Quiroga, Susana Soliño-Lourido, Carmen Pallas-Queijo, Clara González-Formoso, Aurelia Constenla-Castro, Soledad Conde-Freire, Ana Clavería

**Affiliations:** 1 Quality and Research Unit, Health Area of Vigo, Galician Health Service, RedIAPP, Group I-Saúde (Institute of Health Research Galicia Sur), CP 36201 Vigo, Spain; anaclaveriaf@gmail.com; 2 Lérez Health Center, Lugar Porta do Sol s/n, Health Area of Pontevedra, CP 36156 Pontevedra, Spain; aranzacity_sl@hotmail.com (S.S.-L.); mabelconstenla@yahoo.es (A.C.-C.); 3 Vigo Family and Community Medicine and Nursing Teaching Unit, Health Area of Vigo, Galician Health Service, RedIAPP, Group I-Saúde (Institute of Health Research Galicia Sur), CP 36201 Vigo, Spain; maikapallas@gmail.com (C.P.-Q.); clara.gonzalez.formoso@sergas.es (C.G.-F.); 4 Val Miñor Health Center, Avenida Portugal, 91 (A Xunqueira)—A Ramallosa, Health Area of Vigo, CP 36379 Pontevedra, Spain; soledad.conde.freire@sergas.es

**Keywords:** geriatric assessment, elderly, primary care, quality of life, caring

## Abstract

The Manageable Geriatric Assessment (MAGIC) questionnaire, recently developed by a group of European family doctors for multidimensional geriatric assessment in primary care, has not yet been evaluated in clinical practice. The objectives of this study were to translate and adapt it to Spanish and to check the association between the limitations of older adults identified by this questionnaire and their perceived health status assessed by the five-level version of the EuroQol-5D (EQ-5D-5L). First, questionnaire translation, back translation and cognitive test were applied. Then, a cross-sectional observational study was performed in two Spanish health centers Galicia, Spain. Participants were 170 people aged over 75, recruited opportunistically by consecutive case sampling. Anonymous surveys were used to collect data. The MAGIC questionnaire, the EQ-5D-5L scale, age and sex were employed. The visual analog scale of EQ-5D-5L (EQ VAS) was used as the outcome variable. Descriptive and bivariate analyses by sex and outcome variable are presented. The linear regression analysis showed an association with quality of life for daily activities, recognizing people and stress incontinence. As this is associated with quality of life, the MAGIC questionnaire may be useful in primary care and a study to investigate the impact on health with a clinical trial would be worth considering.

## 1. Introduction

The pace of population aging worldwide is dramatically increasing [1]. The number of people aged 60 years and older will increase from 900 million to 2 billion by 2050 [2]. Furthermore, people over the age of 70 will spend an average of 8 years living with disabilities [3]. Although the world is rapidly moving toward an aging population, health systems do not generally fall in line with this trend. Most of the world’s health services have been designed according to acute healthcare models that do not coincide with the main health problems encountered in elderly adults. This healthcare shortcoming is aggravated by discrimination due to age and ignores the elderly’s priorities and requirements [4]. This means having to improve current health services [5].

Geriatric assessments of a preventive, proactive and evidence-based nature can help to promote health and function in the elderly [6]. Most geriatric assessments are tailored to the specific needs of institutionalized individuals and focus on function and cognition. Nevertheless, they rarely adapt to clinical practice in primary care (PC) and to older adults living in the community [7].

Morley’s 2017 review [3] identified and analyzed several tools validated for use in PC to detect health problems by assessing different health domains, and even quality of life: the WHO Disability Scale (WHODAS), the Gérontopôle Fragility Screening Tool (GFST), the Two step Older persons Screening (EASYCare TOS) and the Kihon Checklist (KCL).

Despite their comprehensibility, these questionnaires are rarely used in clinical practice because lack of time makes their implementation difficult [7]. To solve this problem, the Manageable Geriatric Assessment (MAGIC) questionnaire was designed and developed by a European group of family doctors in Germany. MAGIC was developed and published in English. Its principles are to provide a brief feasible geriatric assessment adapted specially to daily PC needs. It consists of nine domains covering health problems and geriatric syndromes: everyday activities, vision, hearing, falls, urinary incontinence, immunization, depression, social support and cognitive impairment. The questionnaire enables the rapid efficient screening of relevant problems related to possible loss of autonomy in the elderly [8].

To date, no study has been carried out on using this tool in Spanish or in clinical practice. As a step prior to study its possible impact on health, we set out to check whether there is a relation between scale items and quality of life.

To this end, the objective of the present study was to translate and adapt the MAGIC questionnaire to Spanish, and to verify the association between older adult limitations identified by the questionnaire and the perceived health status assessed with the EQ VAS.

## 2. Materials and Methods

### 2.1. Design and Location

A cross-sectional observational study was carried out between 2017 and 2018 in health centers in two health areas of Galicia (Spain): Val Miñor in Vigo and Lérez in Pontevedra.

The Spanish National Health System is a system of universal coverage and public financing whose territorial organization is based on Autonomous Communities. In Galicia, with a population of 2.7 million inhabitants, there are 398 primary care centers, each with 2–10 mini-medical/nurse teams that serve an average of 1500 citizens.

### 2.2. Study Population

The study population comprised patients aged 75 or older receiving nursing care in two health centers. Patients with severe cognitive impairment according to the electronic health record (EHC) diagnosis, the inability to speak Spanish, a life expectancy of less than 1 year and/or insufficient reading ability to answer the questionnaire were excluded.

A sample size of 169 was necessary for an expected population of 300 patients in the researchers’ offices, with an expected frequency of 50% in the worst case, accuracy equal to +/−5% and an alpha risk of 95%. The OpenEpi version 3 software was used.

Consecutive sampling with replacement was performed for patient recruitment, and the first three patients attending the office every day and meeting the inclusion criteria were selected. Patients self-completed the questionnaire anonymously and deposited it in an authorized box.

### 2.3. Measurements

For measurements, age, sex and data from the following scales were collected:

- MAGIC questionnaire [8] with nine domains (daily activities, vision, hearing, falls, urinary incontinence, vaccination, depression, social environment and cognition) and 16 items: 15 categorical response items and one item that includes a question with the clock-drawing test, with scores from 1 to 7. Except for the clock-drawing test, the other items did not score (Table 1).

**Figure 1:**
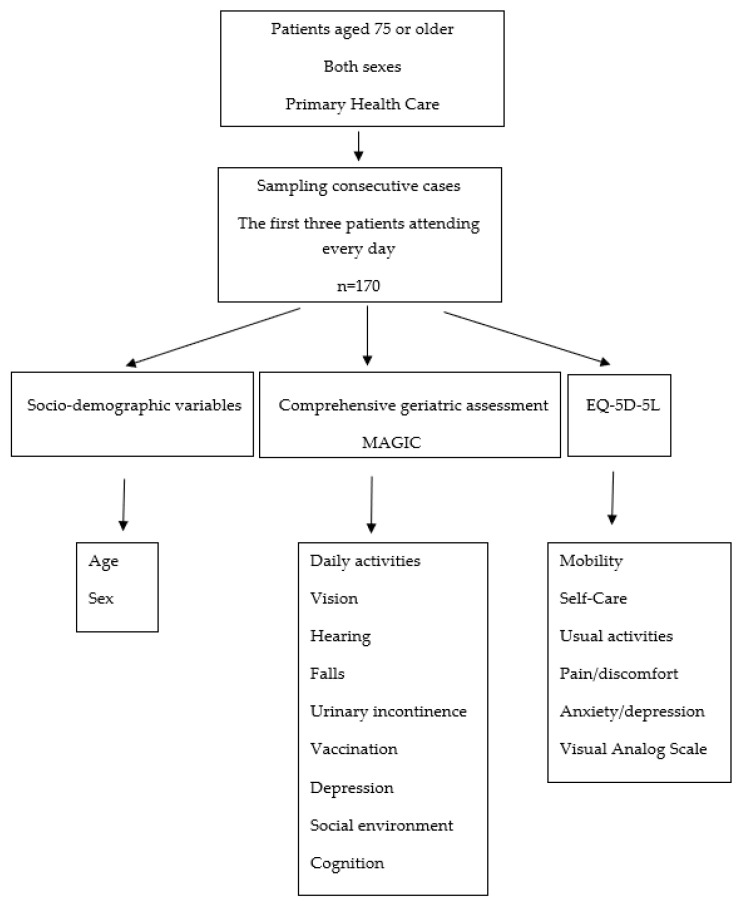
Study of older adults in primary care with multidimensional geriatric assessment.

The study design is presented (Figure 1).

### 2.4. Methods

To adapt MAGIC to our context, the translation and back-translation method was used following the International Society for Pharmacoeconomics and Outcomes Research (ISPOR) methodology [10].

- Direct translation by an official translator was followed by back translation by another professional, with assessment of equivalences following Guillemin [11] and Beaton [12];
- The wordings in some questions (Do you have someone to trust and confide in? In the past month, have you often been bothered by feeling down, depressed or hopeless? In the past month, have you often been bothered by feeling little interest or pleasure in doing things?) were compared to those used in questionnaires validated in Spanish, such as the Older Americans Resources and Services (OARS) Social Resources Scale [13], the Whooley questions [14] and the COOP/WONCA Functional Assessment Charts [15], respectively;
- Unlike the original instrument, with clock scores ranging from 1 to 7 (without specifying how it was quantified), we followed Thalmann’s assessment [16]. This consists of scoring the following items: 1 point if all 12 numbers are present; 2 points if the number 12 is placed correctly; 2 points if hands are correctly proportional; 2 points if the subject is able to tell the time correctly. The optimal cut-off score was 5 points out of a total of 7;
- “Immunization” has been changed from the original MAGIC questionnaire to “vaccine” to facilitate understanding;
- The question on pneumococcal vaccination was included as this is recommended in Galicia (Spain) [17];
- A cognitive test was performed with 10 patients over 75 years of age to check if wording and font size were acceptable.

After the translation and back translation had been completed according to the protocol, the questions about “person to trust”, “depression” and “daily activities” were included using the wording from the validated scales in Spanish [13,14,15].

For the data analysis, a descriptive study of the MAGIC questionnaire and the EQ-5D-5L scale was carried out. Response percentages were calculated for the qualitative variables, and confidence intervals and the median/interquartile range for the quantitative ones. Nonparametric tests were used for the bivariate analysis. Linear regression was performed to analyze the adjusted association of each MAGIC questionnaire item with quality of life (measured by EQ VAS). The relation of the EQ-5D-5L items with the outcome variable was also analyzed in the same way. Automatic data preparation performed by SPSS includes measurement level adjustment, outlier and missing value handling, and supervised merging; categories that are not significantly different (that is, have a *p*-value greater than 0.1) are merged. SPSS v25 was employed.

This study was approved by the Clinical Research Ethics Committee of Galicia (code 2017/497).

## 3. Results

A cognitive test was performed with 10 patients, which did not lead to any modification in the proposed translation. The resulting questionnaire (MAGIC) is presented in Appendix B, Figure A2.

Of the 170 interviewed people, 62.4% with a confidence interval (95% CI) from 54.9 to 69.4 were women and 37.6% (95% CI: 30.6–45.1) were men of a median age of 82 (interquartile range (IQR): 79–85). Three people declined to participate in the study. The time needed to complete both instruments was 15–20 min. The participants answered all the questions (100%), except in the clock-drawing test, which six people did not answer.

The MAGIC results highlight that 15.9% (11.0–21.9) had considerable difficulty in carrying out everyday activities, 71.8% (64.7–78.1) had no difficulties in recognizing people, 72.4% (65.3–78.7) had no falls in the last 6 months, and 98.8% (96.3–99.8) had someone they could trust. In the clock-drawing test, a median of 3 points was obtained with an interquartile range from 1 to 5 (Table 2). Those people who scored below 5 in the clock-drawing test came to 74.4% (67.3–80.6).

On the EQ-5D-5L scale, 60.6% (53.1–67.7) had no problems with bathing or dressing, and 69% (62.1–75.9) reported pain/discomfort at varying degrees of intensity (Table 3).

For the outcome variable (EQ VAS), a median score of 60 was observed with an interquartile range from 50 to 80. There were no significant differences by sex.

In the analysis by sex using MAGIC shown in Appendix C, Table A1, women were more depressed, had more cognitive impairment and more problems with stress and urge incontinence than men. Conversely, men had more hearing problems, but fewer problems with mobility when walking or doing everyday activities, and less anxiety/depression than women.

The bivariate analysis for the outcome variable showed the following to be significant: daily activities, newspaper vision, recognizing people, urge incontinence, depressed, trusted person. The results are detailed in Appendix D, Table A2.

In the linear regression analysis, the MAGIC questionnaire variables associated with quality of life were daily activities, recognizing people and stress incontinence. The variability explained by the model was 20.6% (Table 4).

A sensitivity analysis was run, in which those individuals whose impairment level was over the cut-off obtained similar results.

In the linear regression analysis, the EQ-5D-5L questionnaire variables associated with quality of life were walking mobility, anxiety/depression and pain/discomfort. The variability explained by the model was 25.5% (Table 5).

Accordingly, we considered including the EQ-5D-5L questionnaire items associated with quality of life in the modified MAGIC questionnaire (MAGICm). As walking mobility and anxiety/depression are already included in daily activities and depressed, we added the pain/discomfort item (Appendix E, Figure A3).

## 4. Discussion

The MAGIC questionnaire variables with the strongest impact on quality of life were: daily activities, recognizing people and stress incontinence. These variables, therefore, indicate problems that should be inquired about and acted on as a priority in nursing practice to improve these patients’ quality of life. Furthermore, Table 4 shows that the variables “having someone you can trust and in the event of an emergency” were not significant, but showed a clearly positive tendency in this direction. They could have been significant with a larger sample. In addition, pain came over as having an impact on quality of life and needs to be prioritized. So, we included it in the MAGICm questionnaire.

A systematic review of the scales incorporating patients’ perspectives, and not only the quantification of clinical parameters assessed by professionals, shows that there is currently no instrument that comprehensively covers all the outcomes frequently sought in PC [18].

A comparison of the scales analyzed by Morley [3] revealed that the MAGIC questionnaire covers the largest number of domains as Morley mainly analyzed disability and frailty. The MAGIC questionnaire shares the assessment of everyday activities, cognition and social support with other assessed instruments. It is noteworthy that it would be interesting to assess nutritional status as measured by the WHODAS and the KCL.

In an update of preventive activities regarding older adults, when suspecting frailty, advocates multidimensional clinical assessment or comprehensive geriatric assessment (CGA) in established or more advanced cases, the 2018 recommendations of the Spanish Program for Preventive Activities and Health Promotion (PAPPS) [19] recommend confirming fragility, assessing needs and establishing adequate and individualized intervention plans. The PAPPS recommendations do not propose a specific comprehensive geriatric assessment model but advise that tests in PC should be simple and compatible with patients’ usual practice. Accordingly, our study proposes a quick simple multidimensional geriatric assessment model. PAPPS recommends assessing hearing, vision, falls and cognitive impairment. These items are also included in the MAGIC questionnaire. Moreover, the aforementioned updated recommendations do not mention aspects such as incontinence, depression, among others, which have a marked effect on quality of life.

Cervantes et al. [20] analyzed the health status of older adults in PC based on a comprehensive geriatric assessment made with people aged 60 and older during five PC visits lasting 30–40 min. The variables in common with our study are vision, hearing, urinary incontinence, cognitive impairment, depression and social support. There are differences in the results obtained in both studies (e.g., 54.7% in our study had hearing problems compared to 27.7% in theirs), possibly due to the age difference between the studied populations: their study included participants from the age of 60, while ours starts at the age of 75. The study clearly highlights the need to create systematic health status detection programs in the PC population with timely multidisciplinary interventions by health teams to improve the quality of life of the elderly.

Compared to the Spanish National Health Survey (ENSE) [21], the observed EQ VAS was 60.38 + 24.11 standard deviation (SD), while 58.56 was detected in the ENSE Spain and 58.98 in the ENSE Galicia. The similarity of the percentage distribution between both studies was considered a positive aspect because it suggests that the population was adequately sampled, despite the fact that selection was carried out through health centers rather than being population-based. For bathing and dressing, the respondents in this study aged 85 and older had fewer problems than those in the national study (no problems 40.38% vs. 47.02%, respectively). Regarding walking mobility (no problems 50.14% vs. 33%, respectively) and everyday activities (no problems 60.3% vs. 45.53%, respectively), the respondents aged 75–84 years in the ENSE Spain had fewer problems than those in our study. For the everyday activities’ variable, a high percentage of those surveyed in Spain were incapable of carrying out such activities compared to our study. No major differences appeared in the pain/discomfort comparison. In both age groups, we found that our respondents reported more anxiety/depression than the ENSE Spain respondents. The ENSE study analyzed the following socio-demographical factors: sex, age, social stratum, country of origin, level of education, economic activity and Spanish Autonomous Community. Conversely, the present study analyzed only age and sex because the purpose of the studied questionnaire was the speed with which it is completed to facilitate its use in PC. In the future, it would be interesting to analyze these other socio-demographic factors with the MAGIC questionnaire.

Liu et al. [22] conducted a systematic review about health literacy and defined it as an individual’s ability to obtain and translate knowledge and information to maintain and improve health in a way that is appropriate to both individuals and the system. Our study did not consider this factor, but it would be worth analyzing it in the future with the HLS-EU-Q16 questionnaire [23] for information and given the importance of knowing if the people who completed the questionnaire completely understood it.

Different methodologies have been put forward to assess geriatric scales. Mueller et al. [24] carried out a prospective study with health and diagnostic measures. It concluded that the presented brief assessment tool is a useful appropriate tool for most geriatric syndromes but cannot replace a comprehensive geriatric assessment. Locatelli et al. [25] conducted a prospective study to evaluate the agreement and reliability of a geriatric assessment. These authors concluded that six of the nine geriatric assessment items described in their study had good to excellent reliability and could be safely used. We opted for a cross-sectional study with health-related quality of life as an element to confirm whether or not this is linked with questionnaire items to provide information before assessing its impact on health.

Sabbagh et al. [26] performed a study about the early detection of slight impairment in PC where the current barriers that prevent it from being suitably and accurately detected were identified. They include short visits which, in accordance with the tests that should be done, must last 10 min or less. The “ideal” tool that they proposed must include three critical components: cognitive evaluation by means of tasks to evaluate memory and execution; functional questionnaires; medical history. For its validation, these authors recommend studies being conducted in several languages using representative populations with slight cognitive impairment, dementia and normal cognition. The questionnaire of the present study has the advantage of being quick to complete, which means that its use in PC is feasible. Moreover, this questionnaire includes an evaluative test, the clock-drawing test, which allows us to identify those patients with slight or moderate cognitive impairment who may often be underdiagnosed so that, when they are detected, their problem can be analyzed and the subject can be informed about what interventions can be made. Finally, it was performed only by excluding serious cognitive impairment, which could entail not being able to complete the questionnaire properly, but it included slight and moderate cognitive impairment. We should remember that this study about the MAGIC questionnaire acted as a pilot study to quantify its relation with quality of life and to evaluate if, in the future, it would be interesting to analyze if significant changes in quality of life took place after detecting problems and making interventions [27]. This questionnaire allows us to know patients to detect suspected problems, including cognitive impairment, to be able to subsequently make the appropriate interventions to improve the situation with the healthcare team, including those people presenting slight or moderate impairment.

When implementing the protocol, we found that patients were highly cooperative, facilitated by its brief application. Difficulties were related mainly to drawing the clock, and several patients expressed difficulty in interpreting the wording of this item. Among the limitations, it is noteworthy that only patients from two nursing centers were selected, although the similar prevalence of the dimensions in EQ-5D-5L is indicative of its representativeness of a similar population. Thus, it would be worth extending the sample size and applying this study to other geographical areas. Another study limitation is that people with slight or moderate cognitive impairment might not properly answer the questionnaire. Finally, because the characteristics of cognitive impairment are manifested variably and heterogeneously, the clock-drawing test may not be sufficient to identify all subtypes of cognitive impairment; hence, a larger sample that would guarantee representativeness in different degrees of cognitive impairment would be appropriate.

## 5. Conclusions

After completing the study, we obtained a translated questionnaire, MAGICm, culturally adapted.

We observed that the MAGIC questionnaire was associated with quality of life. In addition, the variables that most strongly impacted quality of life were: daily activities, recognizing people and stress incontinence. These variables, therefore, indicate problems that should be inquired about and acted on as a priority in nursing practice to improve these patients’ quality of life. Given its impact on quality of life, we believe that adding the pain/discomfort question to the initial questions on the MAGICm questionnaire is justified.

As MAGICm is associated with quality of life, a study to investigate the impact on health with a clinical trial would be worth considering to analyze if significant changes in quality of life would take place after detecting problems and making interventions.

## Figures and Tables

**Table 1:** Manageable Geriatric Assessment (MAGIC) questionnaire.

Domains	Items	Response Scale	Scoring	Direction
Daily activities	In the past 2 weeks: how much difficulty have you had doing your usual activities or tasks, both inside and outside the house because of your physical and emotional health?	None, slight, some, considerable or could not do them	No	
Vision	Do you have difficulty seeing newspaper print, even with glasses?	Yes or No	No	
	Do you have difficulty recognizing people across the road, even with glasses?	Yes or No	No	
Hearing	Do you have difficulty hearing a conversation maybe even with a hearing aid?	Yes or No	No	
Falls	Have you had a fall/falls in the last 6 months? How many falls?	Less than 2 or 2 or more	No	
Urinary incontinence	Have you leaked urine when coughing, laughing, running or stooping?	Never, Rarely, Sometimes, Often or Always	No	
	Do you experience any leakage before reaching the toilet?	Never, Rarely, Sometimes, Often or Always	No	
Immunization	Have you had an influenza vaccination in the last 12 months?	Yes, No or Don’t Know	No	
	Have you had a tetanus vaccination in the last 10 years?	Yes, No or Don’t Know	No	
	Have you had a diphtheria vaccination in the last 10 years?	Yes, No or Don’t Know	No	
	Have you had a pneumococcal vaccination in the last 10 years?	Yes, No or Don’t Know	No	
Depression	In the past month, have you often been bothered by feeling down, depressed or hopeless?	Yes or No	No	
	In the past month, have you often been bothered by showing little interest or pleasure in doing things?	Yes or No	No	
Social environment	Do you have someone who would be able to help you in the event of an emergency?	Yes, No or Maybe	No	
	Do you have someone to trust and confide in?	Yes, No or Maybe	No	
Cognition	The clock-drawing test		1–7 points	<5 (Problem) or ≥5 (No Problem)

**Figure A1:**
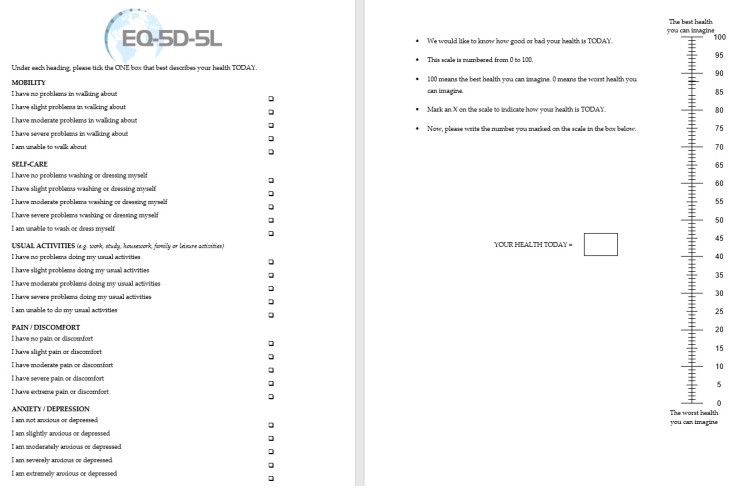
EQ-5D-5L.

**Figure A2:**
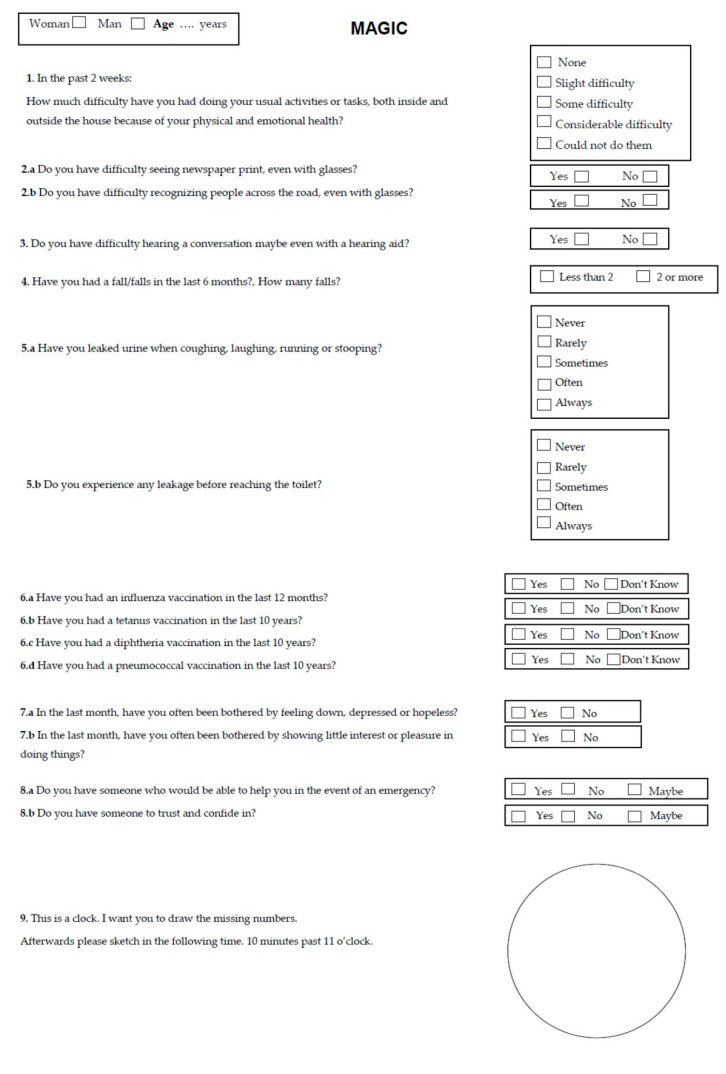
MAGIC.

**Figure A3:**
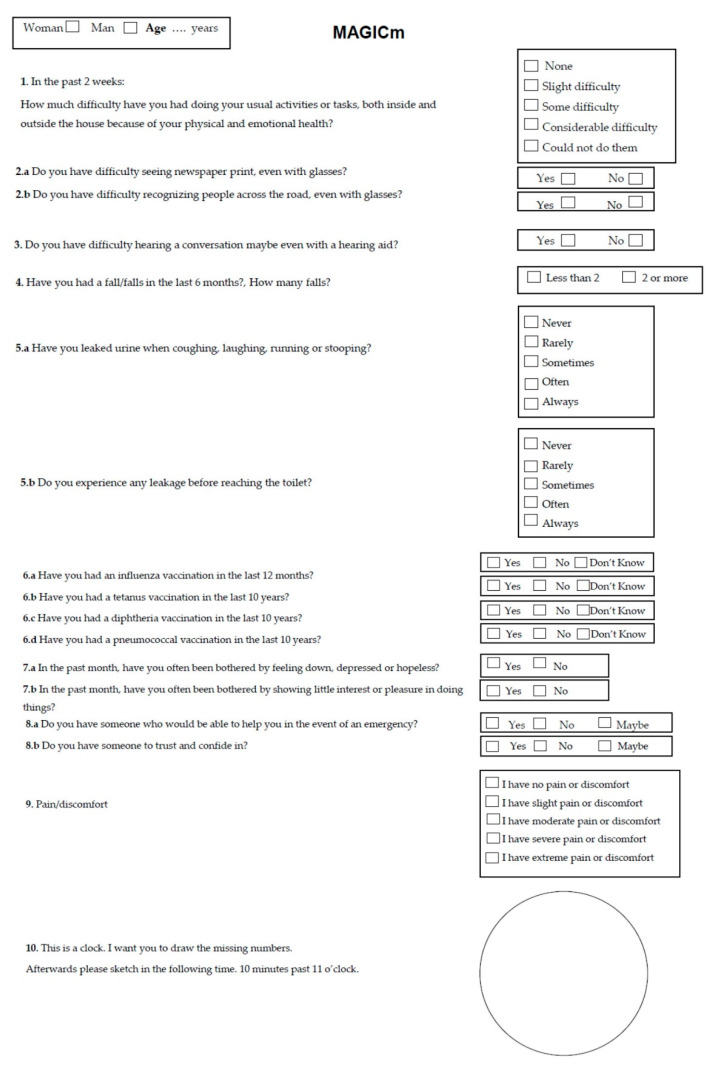
MAGICm.

EQ-5D-5L Scale [9] (Appendix A, Figure A1). Five items, namely mobility, self-care, usual activities, pain/discomfort, anxiety/depression, had five categories each going from less to more (no problem to could not do them). One item is the patients’ own assessment of their health (EQ VAS), (“patients’ personal assessment of their current health” on a scale from 0 to 100). Except for EQ VAS, the five items did not score. EQ VAS was taken as the outcome variable for being a quantitative variable with a foreseeable wide range.

**Table 2:** MAGIC descriptive statistics.

		N (%) N = 170 *	95% CI		
Daily activities	No difficulty	66 (38.8%)	31.7	–	46.3
	A little difficulty	34 (20.0%)	14.5	–	26.5
	Some difficulty	34 (20.0%)	14.5	–	26.5
	Considerable	27 (15.9%)	11.0	–	21.9
	Could not do them	9 (5.3%)	2.7	–	9.4
Newspaper vision	Yes	78 (45.9%)	38.5	–	53.4
	No	92 (54.1%)	46.6	–	61.5
Recognizing people	Yes	48 (28.2%)	21.9	–	35.3
	No	122 (71.8%)	64.7	–	78.1
Hearing	Yes	93 (54.7%)	47.2	–	62.1
	No	77 (45.3%)	37.9	–	52.8
Falls in the last 6 months	Yes	47 (27.6%)	21.3	–	34.7
	No	123 (72.4%)	65.3	–	78.7
Number of falls		1.0	1.0	–	2.0
Stress urinary incontinence	Never	73 (43.5%)	36.1	–	51.0
	Rarely	24 (14.3%)	9.6	–	20.2
	Sometimes	40 (23.8%)	17.9	–	30.7
	Often	20 (11.9%)	7.7	–	17.4
	Always	11 (6.5%)	3.5	–	11.0
Urgency urinary incontinence	Never	67 (39.9%)	32.7	–	47.4
	Rarely	21 (12.5%)	8.1	–	18.1
	Sometimes	43 (25.6%)	19.5	–	32.6
	Often	23 (13.7%)	9.1	–	19.5
	Always	14 (8.3%)	4.9	–	13.2
An influenza vaccination in the last 12 months	Yes	148 (87.1%)	81.4	–	91.5
	No	22 (12.9%)	8.5	–	18.6
	Don’t know	0			
A tetanus vaccination in the last 10 years	Yes	81 (47.6%)	40.2	–	55.1
	No	47 (27.6%)	21.3	–	34.7
	Don’t know	42 (24.7%)	18.7	–	31.6
A diphtheria vaccination in the last 10 years	Yes	75 (44.1%)	36.8	–	51.6
	No	50 (29.4%)	23.0	–	36.6
	Don’t know	45 (26.5%)	20.3	–	33.5
A pneumococcal vaccination in the last 10 years	Yes	76 (44.7%)	37.4	–	52.2
	No	43 (25.3%)	19.2	–	32.2
	Don’t know	51 (30%)	23.5	–	37.2
Depressed in the past month	Yes	94 (55.3%)	47.8	–	62.6
	No	76 (44.7%)	37.4	–	52.2
Little interest doing things	Yes	87 (51.2%)	43.7	–	58.6
	No	83 (48.8%)	41.4	–	56.3
Person to help in an emergency	Yes	156 (91.8%)	86.9	–	95.2
	No	12 (7.1%)	3.9	–	11.6
	Maybe	2 (1.2%)	0.2	–	3.7
Trusted person	Yes	168 (98.8%)	96.3	–	99.8
	No	2 (1.2%)	0.2	–	3.7
	Maybe	0			
Clock-drawing test (Median/IQR)		3	1.0	–	5.0

* Data are numbers (%) and 95% confidence interval or the median and interquartile range (IQR).

**Table 3:** EQ-5D-5L descriptive statistics.

		N (%) N = 170 *	95% CI		
Mobility	No problem	50 (29.4%)	23.0	–	36.6
	Slight	40 (23.5%)	17.6	–	30.3
	Moderate	44 (25.9%)	19.7	–	32.8
	Severe	32 (18.8%)	13.5	–	25.2
	Could not do	4 (2.4%)	0.8	–	5.5
Self-care	No problem	103 (60.6%)	53.1	–	67.7
	Slight	32 (18.8%)	13.5	–	25.2
	Moderate	26 (15.3%)	10.5	–	21.3
	Severe	5 (2.9%)	1.1	–	6.3
	Could not do	4 (2.4%)	0.8	–	5.5
Usual activities	No problem	70 (41.2%)	34.0	–	48.7
	Slight	49 (28.8%)	22.4	–	35.9
	Moderate	32 (18.8%)	13.5	–	25.2
	Severe	15 (8.8%)	5.2	–	13.8
	Could not do	4 (2.4%)	0.8	–	5.5
Pain/discomfort	No problem	52 (30.6%)	24.0	–	37.8
	Slight	48 (28.2%)	21.9	–	35.3
	Moderate	41 (24.1%)	18.2	–	30.9
	Severe	27 (15.9%)	11.0	–	21.9
	Extreme	2 (1.2%)	0.2	–	3.7
Anxiety/depression	No problem	73 (42.9%)	35.7	–	50.4
	Slight	42 (24.7%)	18.7	–	31.6
	Moderate	32 (18.8%)	13.5	–	25.2
	Severe	20 (11.8%)	7.6	–	17.2
	Extreme	3 (1.8%)	0.5	–	4.6
EQ VAS (Median/IQR)		60.0	50.0	–	80.0

* Data are numbers (%) and 95% confidence interval or the median and interquartile range (IQR).

**Table 4:** Linear regression of the MAGIC questionnaire for the outcome variable (EQ VAS) *.

	Coefficient	95% CI			*p*
Interception	13.631	−19.49	–	46.753	0.418
Daily activities = None and a little difficulty.	10.468	3.464	–	17.472	0.004
Daily activities = Some, considerable and could not do them.	0				
Stress urinary incontinence = Never.	13.359	3.789	–	22.930	0.007
Stress urinary incontinence = Rarely and sometimes.	6.217	−3.486	–	15.920	0.208
Stress urinary incontinence = Often and always.	0				
Recognizing people = Yes	−10.014	−17.64	–	−2.393	0.01
Recognizing people = No	0				
Person to help in an emergency = Yes	10.332	−1.804	–	22.468	0.095
Person to help in an emergency = No	0				
Trusted person = Yes	26.097	−5.553	–	57.746	0.105
Trusted person = No	0				

* This coefficient is set at zero because it is redundant.

**Table 5:** Linear regression of the EQ-5D-5L for the outcome variable (EQ VAS) *.

	Coefficient	95% CI			*p*
Interception	29.884	19.341	–	40.43	0.000
Mobility = No problem.	19.658	9.787	–	29.53	0.000
Mobility = Slight and moderate.	12.21	3.617	–	20.8	0.006
Mobility = Severe and could not do.	0				
Anxiety/depression = No problem and slight.	15.329	5.420	–	25.24	0.003
Anxiety/depression = Moderate.	8.044	−3.398	–	19.46	0.167
Anxiety/depression = Severe and extreme.	0				
Pain/discomfort = No problem.	14.248	3.589	–	24.91	0.009
Pain/discomfort = Slight and moderate.	4.665	−4.785	–	14.12	0.331
Pain/discomfort = Severe and extreme.	0				

* This coefficient is set to zero because it is redundant.

## Appendix C

**Table A1:** Bivariate by sex.

		Sex														
		Woman							Man							*p*
		N	%	Lost	Median	IQR *			N	%	Lost	Median	IQR *			
Age		106		0	82.0	79.0	–	85.0	64		0	81.0	77.5	–	85.0	0.268
Daily activities	No difficulty	34	32.1			23.8	–	41.4	32	50.0			38.0	–	62.0	0.130
	Slight difficulty	24	22.6			15.5	–	31.3	10	15.6			8.3	–	25.9	
	Some difficulty	22	20.8			13.9	–	29.2	12	18.8			10.7	–	29.6	
	Considerable	19	17.9			11.5	–	26.0	8	12.5			6.1	–	22.2	
	Could not do them	7	6.6			3.0	–	12.5	2	3.1			0.7	–	9.6	
Newspaper vision	Yes	47	44.3			35.1	–	53.8	31	48.4			36.5	–	60.5	0.611
	No	59	55.7			46.2	–	64.9	33	51.6			39.5	–	63.5	
Recognizing people	Yes	31	29.2			21.2	–	38.4	17	26.6			16.9	–	38.2	0.632
	No	75	70.8			61.6	–	78.8	47	73.4			61.8	–	83.1	
Hearing	Yes	51	48.1			38.8	–	57.6	42	65.6			53.5	–	76.4	0.039
	No	55	51.9			42.4	–	61.2	22	34.4			23.6	–	46.5	
Falls in the last 6 months	Yes	34	32.1			23.8	–	41.4	13	20.3			11.9	–	31.3	0.122
	No	72	67.9			58.6	–	76.2	51	79.7			68.7	–	88.1	
Number of falls		106		72	1.0	1.0	–	2.0	64		51	2.0	1.0	–	2.0	0.680
Stress urinary incontinence	Never	34	32.1			23.8	–	41.4	39	62.9			50.5	–	74.1	0.000
	Rarely	14	13.2			7.8	–	20.6	10	16.1			8.6	–	26.7	
	Sometimes	30	28.3			20.4	–	37.4	10	16.1			8.6	–	26.7	
	Often	18	17.0			10.8	–	25.0	2	3.2			0.7	–	9.9	
	Always	10	9.4			5.0	–	16.1	1	1.6			0.2	–	7.3	
Urgency urinary incontinence	Never	31	29.2			21.2	–	38.4	36	58.1			45.6	–	69.7	0.001
	Rarely	13	12.3			7.1	–	19.5	8	12.9			6.3	–	22.9	
	Sometimes	30	28.3			20.4	–	37.4	13	21.0			12.3	–	32.3	
	Often	21	19.8			13.1	–	28.2	2	3.2			0.7	–	9.9	
	Always	11	10.4			5.6	–	17.2	3	4.8			1.4	–	12.4	
An influenza vaccination in the last 12 months	Yes	92	86.8			79.4	–	92.2	56	87.5			77.8	–	93.9	0.955
	No	14	13.2			7.8	–	20.6	8	12.5			6.1	–	22.2	
	Don’t know	0	0.0				–		0	0.0				–		
A tetanus vaccination in the last 10 years	Yes	50	47.2			37.8	–	56.6	31	48.4			36.5	–	60.5	0.444
	No	33	31.1			22.9	–	40.4	14	21.9			13.1	–	33.1	
	Don’t know	23	21.7			14.7	–	30.2	19	29.7			19.6	–	41.6	
A diphtheria vaccination in the last 10 years	Yes	46	43.4			34.2	–	52.9	29	45.3			33.5	–	57.5	0.689
	No	34	32.1			23.8	–	41.4	16	25.0			15.7	–	36.5	
	Don’t know	26	24.5			17.1	–	33.3	19	29.7			19.6	–	41.6	
A pneumococcal vaccination in the last 10 years	Yes	48	45.3			36.0	–	54.8	28	43.8			32.1	–	56.0	0.242
	No	31	29.2			21.2	–	38.4	12	18.8			10.7	–	29.6	
	Don’t know	27	25.5			17.9	–	34.3	24	37.5			26.4	–	49.7	
Depressed in the past month	Yes	65	61.3			51.8	–	70.2	29	45.3			33.5	–	57.5	0.026
	No	41	38.7			29.8	–	48.2	35	54.7			42.5	–	66.5	
Little interest in doing things	Yes	56	52.8			43.4	–	62.2	31	48.4			36.5	–	60.5	0.449
	No	50	47.2			37.8	–	56.6	33	51.6			39.5	–	63.5	
Person to help in an emergency	Yes	96	90.6			83.9	–	95.0	60	93.8			85.8	–	97.9	0.600
	No	9	8.5			4.3	–	14.9	3	4.7			1.3	–	12.0	
	Maybe	1	0.9			0.1	–	4.3	1	1.6			0.2	–	7.1	
Trusted person	Yes	104	98.1			94.1	–	99.6	64	100.0				–		0.532
	No	2	1.9			0.4	–	5.9	0	0.0				–		
	Maybe	0	0.0				–		0	0.0				–		
Clock-drawing test		106		2	2.0	0.0	–	3.0	64		4	3.0	1.5	–	7.0	0.009
Mobility	No problem	24	22.6			15.5	–	31.3	26	40.6			29.2	–	52.9	0.048
	Slight	26	24.5			17.1	–	33.3	14	21.9			13.1	–	33.1	
	Moderate	29	27.4			19.6	–	36.4	15	23.4			14.4	–	34.8	
	Severe	24	22.6			15.5	–	31.3	8	12.5			6.1	–	22.2	
	Could not do	3	2.8			0.8	–	7.4	1	1.6			0.2	–	7.1	
Self-care	No problem	59	55.7			46.2	–	64.9	44	68.8			56.8	–	79.1	0.302
	Slight	24	22.6			15.5	–	31.3	8	12.5			6.1	–	22.2	
	Moderate	18	17.0			10.8	–	25.0	8	12.5			6.1	–	22.2	
	Severe	3	2.8			0.8	–	7.4	2	3.1			0.7	–	9.6	
	Could not do	2	1.9			0.4	–	5.9	2	3.1			0.7	–	9.6	
Usual activities	No problem	36	34.0			25.5	–	43.3	34	53.1			41.0	–	65.0	0.014
	Slight	33	31.1			22.9	–	40.4	16	25.0			15.7	–	36.5	
	Moderate	21	19.8			13.1	–	28.2	11	17.2			9.5	–	27.8	
	Severe	14	13.2			7.8	–	20.6	1	1.6			0.2	–	7.1	
	Could not do	2	1.9			0.4	–	5.9	2	3.1			0.7	–	9.6	
Pain/discomfort	No problem	27	25.5			17.9	–	34.3	25	39.1			27.8	–	51.3	0.250
	Slight	29	27.4			19.6	–	36.4	19	29.7			19.6	–	41.6	
	Moderate	29	27.4			19.6	–	36.4	12	18.8			10.7	–	29.6	
	Severe	19	17.9			11.5	–	26.0	8	12.5			6.1	–	22.2	
	Extreme	2	1.9			0.4	–	5.9	0	0.0				–		
Anxiety/depression	No problem	36	34.0			25.5	–	43.3	37	57.8			45.6	–	69.3	0.005
	Slight	29	27.4			19.6	–	36.4	13	20.3			11.9	–	31.3	
	Moderate	20	18.9			12.3	–	27.1	12	18.8			10.7	–	29.6	
	Severe	18	17.0			10.8	–	25.0	2	3.1			0.7	–	9.6	
	Extreme	3	2.8			0.8	–	7.4	0	0.0				–		
EQ VAS		106		0	60.0	50.0	–	80.0	64		0	60.0	50.0	–	80.0	0.446

* Interquartile range.

## Appendix D

**Table A2:** The bivariate analysis for the outcome variable.

		N	Median	IQR *			*p*
Daily activities	No difficulty	66	73	50	–	90	0.000
	Slight difficulty	34	60	50	–	70	
	Some difficulty	34	50	35	–	70	
	Considerable	27	50	40	–	60	
	Could not do them	9	50	30	–	70	
Newspaper vision	Yes	78	50	40	–	75	0.005
	No	92	70	50	–	80	
Recognizing people	Yes	48	50	30	–	70	0.000
	No	122	63	50	–	80	
Hearing	Yes	93	60	50	–	80	0.398
	No	77	60	40	–	75	
Falls in the last 6 months	Yes	47	50	35	–	80	0.093
	No	123	60	50	–	80	
Stress urinary incontinence	Never	73	70	50	–	85	0.005
	Rarely	24	60	50	–	80	
	Sometimes	40	55	50	–	78	
	Often	20	50	30	–	60	
	Always	11	50	30	–	80	
Urgency urinary incontinence	Never	67	70	50	–	80	0.049
	Rarely	21	50	50	–	60	
	Sometimes	43	60	50	–	80	
	Often	23	50	30	–	70	
	Always	14	70	30	–	90	
An influenza vaccination in the last 12 months	Yes	148	60	50	–	80	0.402
	No	22	65	50	–	85	
	Don’t know	0	.	.	–	.	
A tetanus vaccination in the last 10 years	Yes	81	60	50	–	80	0.850
	No	47	60	50	–	80	
	Don’t know	42	60	50	–	80	
A diphtheria vaccination in the last 10 years	Yes	75	60	50	–	80	0.880
	No	50	60	50	–	80	
	Don’t know	45	60	50	–	80	
A pneumococcal vaccination in the last 10 years	Yes	76	60	50	–	80	0.440
	No	43	70	50	–	90	
	Don’t know	51	60	50	–	75	
Depressed in the past month	Yes	94	50	40	–	75	0.014
	No	76	65	50	–	80	
Little interest in doing things	Yes	87	55	40	–	80	0.089
	No	83	60	50	–	80	
Person to help in an emergency	Yes	156	60	50	–	80	0.055
	No	12	50	30	–	60	
	Maybe	2	43	35	–	50	
Trusted person	Yes	168	60	50	–	80	0.014
	No	2	15	0	–	30	
	Maybe	0	.	.	–	.	
Mobility	No problem	50	75	60	–	90	0.000
	Slight	40	60	50	–	80	
	Moderate	44	60	50	–	70	
	Severe	32	45	30	–	65	
	Could not do	4	30	30	–	40	
Self-care	No problem	103	60	50	–	80	0.005
	Slight	32	60	50	–	80	
	Moderate	26	50	35	–	80	
	Severe	5	30	20	–	50	
	Could not do	4	35	30	–	45	
Usual activities	No problem	70	70	55	–	80	0.000
	Slight	49	60	50	–	80	
	Moderate	32	50	38	–	70	
	Severe	15	40	30	–	50	
	Could not do	4	30	20	–	40	
Pain/discomfort	No problem	52	80	50	–	90	0.000
	Slight	48	60	50	–	80	
	Moderate	41	60	50	–	65	
	Severe	27	50	30	–	55	
	Extreme	2	30	30	–	30	
Anxiety/depression	No problem	73	60	50	–	80	0.000
	Slight	42	70	50	–	80	
	Moderate	32	50	45	–	70	
	Severe	20	40	30	–	58	
	Extreme	3	35	30	–	40	

* Interquartile range.
